# Efficacy of protocol-based pharmacotherapy management in switching of antibiotic administration routes and dose adjustment based on renal function: a before-after study

**DOI:** 10.1186/s40780-025-00512-8

**Published:** 2025-12-24

**Authors:** Yuki Sato, Yudai Takatani, Shunsaku Nakagawa, Yoshiki Katada, Yasuhiro Tsuchido, Mitsuhiro Sugimoto, Masanori Kimata, Mamiko Saigo, Masahiro Tsuda, Shigeru Ohtsuru, Tomohiro Terada

**Affiliations:** 1 Department of Clinical Pharmacology and Therapeutics, Kyoto University Hospital, Kyoto, Japan; 2 Department of Primary Care and Emergency Medicine, Graduate School of Medicine, Kyoto University, 54 Kawahara-cho, Shogoin, Sakyo-ku, Kyoto, 606-8507 Japan; 3 Department of Infection Control and Prevention, Kyoto University Hospital, Kyoto, Japan; 4 Department of Clinical Laboratory Medicine, Graduate School of Medicine, Kyoto University, Kyoto, Japan

**Keywords:** Protocol-based pharmacotherapy management, Antibiotic stewardship, Dosage adjustment, Renal function, Intravenous-to-oral switch, K-COGaI criteria

## Abstract

**Background:**

Antibiotics are crucial for treating infectious diseases, but their appropriate use is essential to minimize adverse reactions and resistance. This study evaluated the effectiveness of protocol-based pharmacotherapy management (PBPM) based on renal function in adjusting antibiotic dosage and implementing an intravenous-to-oral switch in older patients at high risk of drug-related adverse events. The study compared antibiotic treatment outcomes before and after PBPM implementation at Kyoto University Hospital.

**Methods:**

This before-and-after study included patients aged ≥ 65 years diagnosed with community-acquired pneumonia or uncomplicated pyelonephritis at Kyoto University Hospital. The control group (January to December 2021) received conventional antibiotic treatment, whereas the PBPM group (June 2022 to May 2023) received treatment based on a protocol developed by pharmacists, emergency physicians, and infectious disease specialists. Primary and secondary outcomes included antibiotic administration duration, clinical response, adverse reactions, and costs.

**Results:**

The study included 78 patients (40 control, 38 PBPM). The PBPM group showed a significantly shorter total antibiotic treatment duration (9.6 ± 4.4 vs. 7.5 ± 2.4 days, *P* < 0.05) and intravenous administration duration (7.1 ± 2.8 vs. 5.6 ± 2.2 days, *P* < 0.01) compared with the control group. The PBPM group also demonstrated lower clinical treatment failure rates and reduced incidences of acute kidney injury and alanine aminotransferase elevation. Antibiotic costs per patient were 13% lower in the PBPM group. There were no significant differences in early clinical response, readmission rates, or mortality between groups.

**Conclusions:**

PBPM, conducted collaboratively by pharmacists and physicians, effectively optimized antibiotic use, reduced adverse event risks, shortened treatment duration, and lowered healthcare costs without compromising clinical outcomes. These findings support PBPM implementation to improve antibiotic stewardship in older patients with common infections.

**Clinical trial number:**

Not applicable.

**Supplementary Information:**

The online version contains supplementary material available at 10.1186/s40780-025-00512-8.

## Background

Antibiotics are essential for the treatment of infectious diseases, and their efficacy is widely recognized. However, appropriate use of these drugs is crucial for minimizing adverse reactions and the emergence of resistance [1, 2]. Therefore, maximizing the efficacy of antibiotics while minimizing their side effects, specifically toxicity and the emergence of resistance, is recommended [3]. In addition, many patients hospitalized with infectious diseases receive antibiotics intravenously (IV), which is more expensive than oral administration (PO) and also incurs indirect costs associated with preparation and administration [4]. Indirect costs have been reported to exceed the cost of the drug itself by 13–113% [5]. One approach to optimize antibiotic use is dose adjustment based on the pharmacokinetics (PK)/pharmacodynamics (PD) theory for IV antibiotics. The second approach is switching from IV to PO administration as soon as the patient is clinically stable. Such interventions, including dose adjustments and changes in administration routes, have been reported to have multiple benefits, including fewer adverse reactions, reduced risk of infection, shorter hospital stays, and reduced associated costs [4, 6–13]. In particular, several randomized controlled trials have shown that early switching to oral antibiotics is safe while maintaining efficacy in common infections such as pneumonia, uncomplicated pyelonephritis, and intra-abdominal infections [7, 14, 15]. Therefore, when using antibiotics, a prompt transition from IV to PO is strongly recommended once patients are clinically stable. This recommendation is outlined in the 2007 American Thoracic Society and Infectious Diseases Society of America (IDSA) Community-Acquired Pneumonia Guidelines and the 2016 IDSA Antibiotic Stewardship Guidelines [16, 17].

Cooperation by a multidisciplinary team is essential in promoting appropriate antibiotic use, and the intervention of pharmacists is particularly effective. The IDSA and the Society for Healthcare Epidemiology of America recommend that pharmacists play an active role in antibiotic stewardship programs as part of their routine practice [16]. In particular, pharmacist-initiated intravenous-to-oral switch (IVOS) after symptom improvement has been reported to be beneficial, leading to shorter antibiotic treatment durations and reduced medical costs [6]. Furthermore, it would be clinically useful for pharmacists to intervene comprehensively, not only by initiating IVOS but also by adjusting antibiotic dosages based on the PK/PD theory. However, no examples of such practices or their clinical significance have been reported thus far. In addition, depending on local regulations, it may be difficult for pharmacists to adjust the antibiotic dosage independently or on behalf of physicians; therefore, establishing predefined protocols that enable pharmacists to intervene in antibiotic treatment would be beneficial. At Kyoto University Hospital, doctors and pharmacists have collaborated to implement protocol-based pharmacotherapy management (PBPM) for antibiotic treatment in emergency patients. This approach is expected to provide safe drug therapy while maintaining clinical outcomes. In this study, we retrospectively examined the effectiveness of PBPM in adjusting antibiotic dosages and implementing IVOS based on renal function in older patients at high risk of drug-related adverse events, including renal dysfunction.

## Methods

### Study design

This before-and-after study aimed to evaluate the efficacy of PBPM in antibiotic treatment. As PBPM was implemented for antibiotic treatment at Kyoto University Hospital in January 2022, we compared older patients who received antibiotic treatment before and PBPM initiation. The control group comprised patients treated from January 2021 to December 2021, and the PBPM group comprised patients treated from June 2022 to May 2023. The period from January to May 2022 was excluded because it represented a transition period before the implementation of PBPM.

### Study participants

The study participants were patients aged 65 years or older diagnosed with community-acquired pneumonia or uncomplicated pyelonephritis during the study period, admitted to the emergency intensive care unit or ward in the Department of Primary Care and Emergency Medicine at Kyoto University Hospital, and initiated IV antibiotic therapy. Patients with bacteremia often require longer treatment periods; therefore, they were excluded to ensure comparable conditions. In addition, patients hospitalized due to complicated infections (e.g., empyema, lung abscess, renal abscess, perinephric abscess), those who completed IV antibiotic treatment within 48 h of initiation, those who began antibiotic treatment before hospitalization, and those transferred to another ward or hospital before completing antibiotic treatment were also excluded.

### Antibiotic treatment using PBPM

When implementing PBPM, pharmacists, emergency physicians, and infectious disease physicians created a protocol for adjusting antibiotic dosages based on renal function (Supplementary Tables 1 and 2), referencing The Sanford Guide to Antimicrobial Therapy (https://www.sanfordguide.com/). Additionally, referencing the COMS criteria—guidelines for the IVOS of antibiotic therapy reported by the University of Nottingham in 2010—a flowchart for performing IVOS tailored to the clinical setting in Japan was created, which we named the K-COGaI criteria (Supplementary Fig. 1). Within PBPM, doctors selected the initial antibiotics for each patient and asked pharmacists to adjust the dosage based on renal function and evaluate IVOS eligibility. Considering the patient’s age, physical constitution, renal function, and the severity of the infection, the pharmacist proposed an antibiotic prescription (including dosage, volume of dissolving solution, infusion time, and administration route) based on the protocols (Supplementary Tables 1 and 2 and Supplementary Fig. 1). The pharmacist regularly reviewed the blood test results and proposed dosage adjustments when necessary. In addition, when a patient receiving antibiotics met all K-COGaI criteria, the pharmacist recommended IVOS to the attending physician. In either case, the pharmacist’s proposal was recorded in the electronic medical record and shared with the doctors and nurses. During weekday shifts (8:30–17:15), pharmacists proactively proposed antibiotic dosage adjustments and IVOS. At other times, physicians determined the dosage and timing of IVOS by referring to the dosage adjustment protocol based on renal function. Patients in the control group received antibiotics before the protocol was established, and the antibiotic dosage and timing of IVOS were determined based on the emergency physician’s knowledge and experience.

### Outcomes

The primary endpoint was the duration of antibiotic administration (days). This duration was defined as the total number of days the patient received systemic antibiotic therapy, including both IV and PO administration, irrespective of whether the administration occurred during hospitalization or after discharge. The secondary endpoints were early clinical response (ECR), clinical treatment failure rate, readmission rate within 28 days after discharge, 28-day mortality, length of hospital stay, rate of adverse reactions, antibiotic cost, and compliance with the dosage adjustment protocol based on renal function. ECR was defined as meeting all of the following three criteria: (i) no clinical deterioration or death within 96 h; (ii) a decrease of ≥ 30% in white blood cell count or C-reactive protein compared with baseline; and (iii) a decrease in body temperature of 0.3 °C for 2 consecutive days from treatment initiation [18]. Clinical treatment failure was defined as a change in broad-spectrum antibiotic use [19] or relapse [20, 21]. Adverse reactions were defined as grade 1 or higher events that occurred during antibiotic use. Acute kidney injury (AKI) was assessed according to the Kidney Disease Improving Global Outcomes clinical practice guidelines [22]. Elevations in aspartate aminotransferase (AST) or alanine aminotransferase (ALT) and diarrhea were assessed according to the Common Terminology Criteria for Adverse Events version 5.0. Compliance with the dosage adjustment protocol based on renal function was calculated as the proportion of patients who adhered to the protocol (Supplementary Tables 1 and 2).

### Other measurements

Causative bacteria detected in sputum and urine were identified by an infectious disease specialist. Information on other variables, including age, sex, and reasons why IVOS could not be performed, was collected from electronic medical records.

### Statistical analysis

Fisher’s exact test was used to compare nominal variables between the two groups, and Student’s t-test was used to compare continuous variables. A risk level of ≤ 5% (*P* < 0.05) was considered statistically significant. GraphPad PRISM (GraphPad Software Inc., San Diego, CA) was used for the analysis.

## Results

### Patients’ demographic and clinical characteristics

**Fig. 1:**
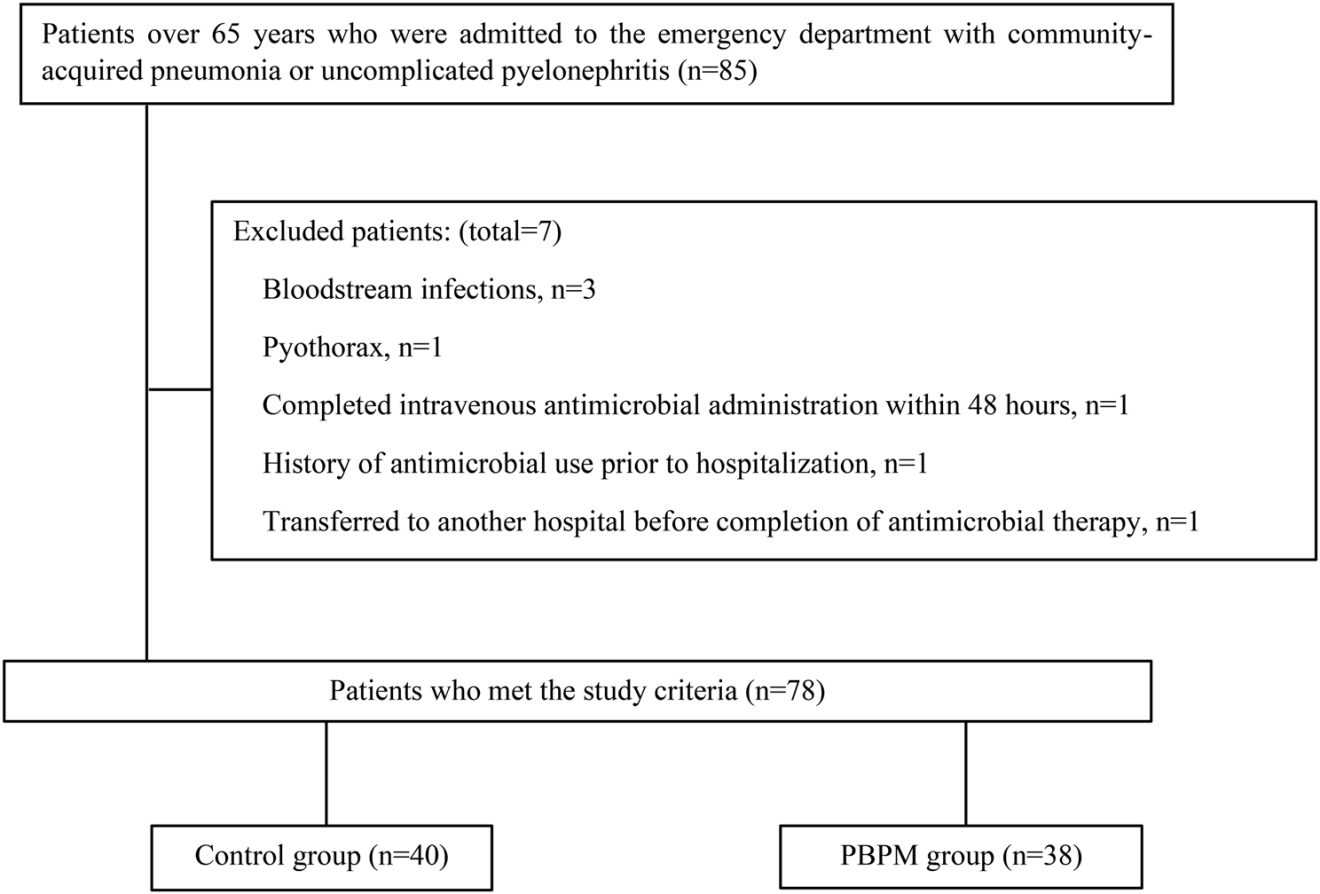
Patient flow diagram. Protocol-based pharmacotherapy management: PBPM

Among 85 patients admitted to the emergency department with community-acquired pneumonia or uncomplicated pyelonephritis, 78 were included in the final analysis (40 control, 38 PBPM) (Fig. 1). Seven patients were excluded for the following reasons: (i) admitted due to bacteremia (*n* = 3), (ii) admission due to empyema (*n* = 1), (iii) completion of IV antibiotics within 48 h of initiation (*n* = 1), (iv) initiation of antibiotics before admission (*n* = 1), and (v) transfer to another ward or hospital before completion of antibiotic treatment (*n* = 1). There were no significant differences between the two groups regarding their demographic and clinical characteristics (Table 1).

**Table 1:** Patients’ demographic and clinical characteristics

	Control group (*n* = 40)	PBPM group (*n* = 38)
Sex, female, n (%)	19 (47.5)	16 (42.1)
Age (years), mean ± SD	83.9 ± 9.1	82.1 ± 14.6
Body weight (kg), mean ± SD	52.6 ± 12.1	54.4 ± 14.4
Creatinine clearance (mL/min), mean ± SD	34.6 ± 23.9	39.5 ± 26.5
CRP (mg/dL), mean ± SD	12.1 ± 7.7	10.6 ± 8.3
WBC (×10^3^/µL), mean ± SD	12.3 ± 5.3	11.5 ± 5.4
Body temperature (℃), mean ± SD	37.8 ± 0.9	37.9 ± 0.7
Underlying disease, n (%)		
Diabetes mellitus	14 (35.0)	16 (42.1)
Ischemic heart disease	7 (17.5)	12 (31.6)
Chronic respiratory disease	1 (2.5)	1 (2.6)
Malignant tumor	0 (0)	4 (10.5)
Others	19 (47.5)	18 (47.4)
Charlson Comorbidity Index, mean ± SD	6.0 ± 1.4	6.1 ± 2.2
Pitt Bacteremia Score, mean ± SD	1.3 ± 1.7	1.9 ± 1.8
Type of infection, n (%)		
Pneumonia	25 (62.5)	28 (73.7)
Pyelonephritis	15 (37.5)	10 (26.3)
Causative organisms in pneumonia, n (%) *		
*Haemophilus influenzae*	1 (4.0)	0 (0)
*Klebsiella pneumoniae*	4 (16.0)	1 (3.6)
*Moraxella catarrhalis*	0 (0)	2 (7.1)
*Pseudomonas aeruginosa*	3 (12.0)	0 (0)
MSSA	1 (4.0)	2 (7.1)
MRSA	1 (4.0)	0 (0)
Others	2 (8.0)	1 (3.6)
Unknown	15 (60.0)	23 (82.1)
Causative organisms in uncomplicated pyelonephritis, n (%) *		
*Enterobacter cloacae complex*	2 (13.3)	0 (0)
*Escherichia coli*	9 (60.0)	7 (70.0)
*Klebsiella oxytoca*	1 (6.7)	1 (10.0)
*Klebsiella pneumoniae*	2 (13.3)	1 (10.0)
*Pseudomonas aeruginosa*	1 (6.7)	0 (0)
*Proteus mirabilis*	1 (6.7)	0 (0)
Others	3 (20.0)	3 (30.0)
Unknown	1 (6.7)	0 (0)
Empiric therapy, n (%)		
Ampicillin/Sulbactam	16 (40.0)	14 (36.8)
Piperacillin/Tazobactam	6 (15.0)	8 (21.1)
Ceftriaxone	12 (30.0)	14 (36.8)
Meropenem	3 (7.5)	2 (5.3)
Others	3 (7.5)	0 (0)

Data are expressed either as means ± standard deviations or numbers with percentages in parentheses. Continuous variables were compared using Student’s t-test. Categorical variables were compared using Fisher’s exact test. *One patient can have more than two indications. Highest value within 24 h of admission. Creatinine clearance was calculated using the Cockcroft–Gault formula. Protocol-based pharmacotherapy management: PBPM, standard deviation: SD, C-reactive protein: CRP, white blood cell: WBC, Methicillin-susceptible *Staphylococcus aureus*: MSSA, Methicillin-resistant *Staphylococcus aureus*: MRSA

### Association between IVOS and PBPM

The proportion of patients who underwent IVOS was 42.5% (17/40) and 39.5% (15/38) in the control and PBPM groups, respectively, with no significant difference (Table 2). Similarly, when categorized into pneumonia and uncomplicated pyelonephritis, there was no significant difference between the groups in the proportion of patients who underwent IVOS. However, when IVOS was performed, the time to switch to oral antibiotics was 6.1 ± 2.3 days (mean ± standard deviation) and 4.2 ± 1.1 days in the control and PBPM groups, respectively, significantly shorter in the PBPM group. In the control group, two patients returned to IV administration after switching to oral antibiotics, whereas no such cases occurred in the PBPM group. Reasons for not performing IVOS included lack of clinical improvement, absence of oral antimicrobial agents effective against the causative bacteria, inability to administer oral antibiotics due to gastrointestinal bleeding or other reasons, and patient intubation (Supplementary Table 3).

**Table 2:** Effect of PBPM on intravenous-to-oral switch

	Control group (*n* = 40)	PBPM group (*n* = 38)
Cases switched from intravenous to oral treatment, n (%)	17 (42.5)	15 (39.5)
Pneumonia	7 (28.0)	7 (25.0)
Uncomplicated pyelonephritis	10 (66.7)	8 (80.0)
Time to switch (days), mean ± SD	6.1 ± 2.3	4.2 ± 1.1**

Data are expressed either as means ± standard deviations or numbers with percentages in parentheses. Continuous variables were compared using Student’s t-test. Categorical variables were compared using Fisher’s exact test. **P < 0.01, significantly different from the control group. Protocol-based pharmacotherapy management: PBPM, standard deviation: SD

### Antibiotic administration duration in the control and PBPM groups

The antibiotic administration duration in the control and PBPM groups is shown in Table 3. The total antibiotic treatment duration (regardless of administration route) was 9.6 ± 4.4 days and 7.5 ± 2.4 days in the control and PBPM groups, respectively, with the PBPM group showing a significant reduction in treatment duration. The duration of IV administration was also significantly reduced in the PBPM group compared to the control group (control, 7.1 ± 2.8 days; PBPM, 5.6 ± 2.0 days). The duration of PO was not significantly different between the two groups (control, 6.1 ± 4.2 days; PBPM, 4.8 ± 2.4 days). Regarding pneumonia alone, there was a significant difference between the two groups in the total antibiotic treatment period (control, 8.8 ± 4.4 days; PBPM, 6.9 ± 2.2 days), and in the duration of IV administration (control, 7.3 ± 3.1 days; PBPM, 5.8 ± 2.2 days), but no significant difference in the duration of PO administration (control, 5.6 ± 4.3 days; PBPM, 4.4 ± 2.4 days). When limited to uncomplicated pyelonephritis, the IV administration period (control, 6.7 ± 2.1 days; PBPM, 4.9 ± 1.5 days) was significantly different between the two groups, but there was no significant difference in the total antibiotic treatment period (control, 10.9 ± 4.3 days; PBPM, 9.0 ± 2.5 days) or in PO duration (control, 6.4 ± 4.3 days; PBPM, 5.1 ± 2.6 days).

**Table 3:** Effect of PBPM on the duration of antibiotic treatment

Total patients	Control group (*n* = 40)	PBPM group (*n* = 38)
Days of intravenous plus oral treatment	9.6 ± 4.4	7.5 ± 2.4*
Days of intravenous treatment	7.1 ± 2.8	5.6 ± 2.2**
Days of oral treatment	6.1 ± 4.2	4.8 ± 2.4

Patients with pneumonia	Control group (*n* = 25)	PBPM group (*n* = 28)
Days of intravenous plus oral treatment	8.8 ± 4.4	6.9 ± 2.2*
Days of intravenous treatment	7.3 ± 3.1	5.8 ± 2.2*
Days of oral treatment	5.6 ± 4.3	4.4 ± 2.4

Patients with uncomplicated pyelonephritis	Control group (*n* = 15)	PBPM group (*n* = 10)
Days of intravenous plus oral treatment	10.9 ± 4.3	9.0 ± 2.5
Days of intravenous treatment	6.7 ± 2.1	4.9 ± 1.5*
Days of oral treatment	6.4 ± 4.3	5.1 ± 2.6

Data are expressed as means ± standard deviations. Continuous variables were compared using Student’s t-test. *P < 0.05, **P < 0.01, significantly different from the control group. Protocol-based pharmacotherapy management: PBPM

### Secondary outcomes in the control and PBPM groups

The secondary outcomes are listed in Table 4. Among the treatment outcomes, the clinical treatment failure rate was significantly lower in the PBPM group than in the control group. There were no significant differences between the two groups in terms of ECR, rehospitalization rate, 28-day mortality, or length of hospital stay. A comparison of the incidence of antibiotic-related adverse events during antibiotic treatment showed that the incidences of AKI and ALT elevation were significantly lower in the PBPM group than in the control group. There were no significant differences in the incidences of AST elevation, diarrhea, or *Clostridioides difficile* infection between the two groups. The total antibiotic costs were 522,105 yen (13,517 yen per patient) and 457,205 yen (11,787 yen per patient) for the control and PBPM groups, respectively. Therefore, the cost per patient was 13% lower in the PBPM group than in the control group. A comparison of the adherence rates to the antibiotic dosage adjustment protocol based on renal function before and after PBPM implementation showed that they were 77.5% (31/40) and 97.4% (37/38) in the control and PBPM groups, respectively, with a significantly higher rate in the PBPM group.

**Table 4:** Secondary outcomes for antibiotic treatment

	Control group (*n* = 40)	PBPM group (*n* = 38)
Treatment success, n (%)		
Early clinical response	18 (45.0)	23 (60.1)
Clinical treatment failures	9 (22.5)	2 (5.6)*
Readmission within 1 month	2 (5.0)	0 (0)
28-day mortality, n (%)	0 (0)	0 (0)
Length of hospital stay (days), mean ± SD	10.4 ± 4.7	10.6 ± 6.2
Adverse events, n (%)		
Nephrotoxicity ^a^	6 (15.0)	0 (0)*
Hematologic abnormality ^b^	0 (0)	0 (0)
Hepatobiliary abnormality ^b^	13 (32.5)	4 (10.5)*
Aspartate aminotransferase increased	11 (27.5)	4 (10.5)
Alanine aminotransferase increased	9 (22.5)	2 (5.3)*
Total bilirubin increased	0 (0)	0 (0)
Non-*Clostridioides difficile*-associated diarrhea	10 (25.0)	14 (36.8)
*Clostridioides difficile* infection	0 (0)	0 (0)
Cost of antibiotics (yen), mean ± SD	13,517 ± 10,462	11,787 ± 6,798
Compliance with renal dosage adjustment protocol for antibiotics		
Yes, n (%)	31 (77.5)	37 (97.4)*
No (overdose), n (%)	3 (7.5)	1 (2.63)
No (underdose), n (%)	6 (15.0)	0 (0)*

Data are expressed either as means ± standard deviations or numbers with percentages in parentheses. Continuous variables were compared using Student’s t-test. Categorical variables were compared using Fisher’s exact test. ^a^ Classified according to the Kidney Disease Improving Global Outcomes (KDIGO) clinical practice guidelines. ^b^ Classified according to the Common Terminology Criteria for Adverse Events (CTCAE) version 5.0 clinical practice guidelines. *P < 0.05, significantly different from the control group. Protocol-based pharmacotherapy management: PBPM, standard deviation: SD

## Discussion

This study examined the effectiveness of PBPM in implementing IVOS and antibiotic dose adjustment based on renal function. The results showed that PBPM shortened the duration of IV antibiotic administration, reduced the risk of adverse events, and lowered pharmaceutical costs without negatively affecting clinical outcomes compared to conventional treatment mainly performed by physicians. The effectiveness of IVOS in PBPM is consistent with the results of a previous study that evaluated the usefulness of IVOS of antibiotics using Collaborative Drug Therapy Management [23]. However, this is the first study to apply a protocol that standardizes the process of IVOS and antibiotic dose adjustment based on renal function, offering advantages different from those of existing studies. The main reason this study targeted older people (aged 65 years and older) is that this population has the highest risk of drug-related adverse events (especially renal dysfunction) due to age-related declines in renal function. Consequently, most older patients require dose adjustments based on renal function. Therefore, we hypothesized that the clinical priority and potential benefit of the PBPM intervention would be greatest in this high-risk population. While PBPM may offer some benefit to younger patients, the specific effects in those under 65 years are outside the scope of this study and cannot be determined from the present findings.

No differences were observed between the control and PBPM groups in ECR, 28-day mortality, readmission within 1 month, proportion of *Clostridioides difficile* infection cases, and length of hospitalization. In addition, during the period in which PBPM was implemented, there were no deaths within 28 days, no rehospitalizations within 1 month, and no cases of *Clostridioides difficile* infection, suggesting that the implementation of PBPM does not worsen clinical outcomes. A meta-analysis reported that IVOS was as effective as continued IV administration in terms of clinical outcomes in community-acquired pneumonia [10]. Therefore, the antimicrobial stewardship program recommended by the IDSA and Society for Healthcare Epidemiology of America strongly recommends that IV antibiotics used in initial treatment should be switched to oral antibiotics at the appropriate time [16]. Therefore, in the case of uncomplicated infections, it is clinically appropriate to actively switch the route of antibiotic administration via PBPM.

The implementation of PBPM by pharmacists in this study was shown to be an effective method for shortening both the total and IV antibiotic administration periods. The duration of IV antibiotic administration was also shortened across different types of infections. There was a difference in the rate of IVOS between patients with pneumonia and those with uncomplicated pyelonephritis. This may be due to the high risk of aspiration and difficulty with oral intake in patients with pneumonia, which may have led to a slower switch to PO (Supplementary Table 3). Nevertheless, the implementation of PBPM could still lead to an appropriate treatment duration, even in patients with limited options. This study, however, did not find any association between the implementation of PBPM and a shortened length of hospital stay. Many patients had difficulty being discharged home; some were not immediately discharged after switching to PO, while others underwent a period of observation after switching. Although future larger studies are needed to confirm its validity, this study suggests that the implementation of PBPM contributes to a shortened antibiotic administration period in both patients with community-acquired pneumonia and uncomplicated pyelonephritis.

Adverse events associated with antibiotic administration occurred in 22.3% of patients, and the risk of adverse events increased by 3% for every 10 days of antibiotic use [24, 25]. Therefore, regular reviews of the dosage and necessity of antibiotics are expected to reduce the risk of adverse events. In this study, the rates of AKI and ALT elevation during the antibiotic administration period significantly decreased after the implementation of PBPM compared to before its implementation. Although compliance with the antibiotic dosage adjustment protocol based on renal function improved after the implementation of PBPM, the rate of overdosing remained unchanged. However, the significant reduction in adverse events can be attributed not only to the shortened administration period but also to the composite nature of the PBPM intervention, specifically the early implementation of IVOS and improved adherence to appropriate dose adjustments. We hypothesize that enhanced adherence to the dose adjustment protocol minimized the risk of toxicity related to over-exposure, particularly in the older patient cohort. Furthermore, the significant reduction in clinical treatment failure observed in the PBPM group is likely due to dose optimization, which reduces both the risk of toxicity from over-dosing and the risk of sub-therapeutic levels, thereby improving the clinical outcomes.

This study had some limitations. First, the patients in the PBPM and control groups were admitted at different times, and the before-after nature of the study introduces potential confounding effects. Specifically, the presence of unmeasured confounding factors cannot be completely ruled out, and it is difficult to quantitatively assess their contribution to the observed outcomes. For instance, the increased knowledge and ability of doctors and pharmacists due to the establishment of the dosage adjustment protocol and IVOS flowchart for PBPM may have indirectly influenced the results. Second, PBPM was implemented primarily during regular weekday operating hours. This non-uniformity of coverage suggests that the absence of pharmacist intervention on weekends and holidays may have contributed to delays in IVOS. In addition, this study was conducted at a single institution with a small sample size; therefore, caution is required when generalizing the effectiveness of PBPM.

## Conclusions

The results of this study suggest that PBPM, when conducted collaboratively by pharmacists and physicians, is an effective method to optimize antibiotic use, reduce the risk of adverse events, and shorten treatment duration, thereby lowering healthcare costs. We believe that these findings will contribute to the improvement of guidelines for the appropriate use of antibiotics. It is also important to evaluate the effectiveness of PBPM in a wider range of patient groups and infectious diseases and to expand its scope of application in future studies. When introducing and implementing PBPM, it is necessary to develop protocols that consider local regulations and restrictions and ensure their appropriate application.

## Supplementary Information

Below is the link to the electronic supplementary material.

Supplementary Material 1

Supplementary Material 2

## Data Availability

The datasets used and/or analyzed during the current study are available from the corresponding author on reasonable request.

## Abbreviations

AKI: Acute kidney injury
ALT: Alanine aminotransferase
AST: Aspartate aminotransferase
ECR: Early clinical response
IDSA: Infectious Diseases Society of America
IV: Intravenous
IVOS: Intravenous-to-oral switch
PBPM: Protocol-based pharmacotherapy management
PD: Pharmacodynamics
PK: Pharmacokinetics
PO: Oral administration
