# Supplementary material for: Efficacy of protocol-based pharmacotherapy management in switching of antibiotic administration routes and dose adjustment based on renal function: a before-after study
Source: J Pharm Health Care Sci. 2025 Dec 24;11:113. doi: 10.1186/s40780-025-00512-8 (PMC12729801; doi:10.1186/s40780-025-00512-8)
Supplement: Supplementary file 1 — Supplementary Material 1 [file 40780_2025_512_MOESM1_ESM.docx]

Supplementary Table 1. Dosage of intravenous antibacterial agents administered to patients with renal impairment

| Drug | | Ccr > 50 mL/min | Ccr 25-50 mL/min | Ccr 10-25 mL/min | Ccr < 10 mL/min | HD |
| --- | --- | --- | --- | --- | --- | --- |
| Penicillin G  (PCG) | | 2-4 million units q4h | 2-4 million units q8h | | 2-4 million units q12h | 2-4 million units q12h* (Post-dialysis administration on dialysis days) |
| Ampicillin  (ABPC) | Usual | 2 g q6h  (Over 30 minutes) | 30-50: 2 g q8h | 10-30: 2 g q12h | 1-2 g q12h | 1-2 g q12h* (Post-dialysis administration on dialysis days) |
|  | Target:  central nervous system/ infective endocarditis | 2 g q4h  (Over 30 minutes) | 30-50: 2 g q6h | 10-30: 2 g q8h | 2 g q12h | 2 g q12h* (Post-dialysis administration on dialysis days) |
| Ampicillin/sulbactam  (ABPC/SBT) | | > 60: 3 g q6h (Over 30 minutes) | 30-60: 3 g q8h | 10-30: 3 g q12h | 3 g q24h | 3 g q24h (Post-dialysis administration on dialysis days) |
| Piperacillin/  tazobactam (PIPC/TAZ) | Usual | > 40: 4.5 g q8h (Over 30 minutes) | 20-40: 2.25 g q6h | < 20: 2.25 g q8h | | 2.25 g q12h* |
|  | Target:  *Pseudomonas aeruginosa* | > 40: 4.5 g q6h (Over 30 minutes) | 20-40: 4.5 g q8h | < 20: 2.25 g q6h | | 2.25 g q8h* |
| Cefazolin (CEZ) | | 1-2 g q8h (Over 30 minutes) | 1-2 g q12h | | 1-2 g q24h | 1-2 g q24h (Post-dialysis administration on dialysis days) |
| Cefmetazole (CMZ) | | > 75: 1 g  q6h (For an hour) | 50-75: 1 g  q8h | 10-50: 1 g  q12h | < 10: 1 g  q 24-48h | 1 g q 24-48h (Post-dialysis administration on dialysis days) |
| Ceftriaxone  (CTRX) | Usual | 1-2 g q24h (Over 30 minutes) | | | | |
|  | Target:  central nervous system infection | 2g q12h (Over 30 minutes) | | | | |
| Cefepime  (CFPM) | Usual | > 60: 2 g q12h (Over 30 minutes) | 30-60: 2 g  IV q24h | 10-30: 1 g  IV q24h | 0.5 g  IV q24h | 0.5 g q24h (Post-dialysis administration on dialysis days) |
|  | Target:  Central nervous system infection | > 60: 2 g q8h (Over 30 minutes) | 30-60: 2 g q12h | 10-30: 1 g q12h | 1 g q24h | 1 g q24h (Post-dialysis administration on dialysis days) |
| Meropenem  (MEPM) | Usual | 1 g q8h (Over 30 minutes) | 1 g q12h | 0.5 g q12h | 0.5 g q24h | 0.5 g q24h (Post-dialysis administration on dialysis days) |
|  | Target:  Central nervous system infection | 2 g q8h (Over 30 minutes) | 2 g q12h | 1 g q12h | 1 g q24h | 1 g q24h (Post-dialysis administration on dialysis days) |
| Ciprofloxacin  (CPFX) | Usual | - 30: 400 mg q12h (For an hour) | | < 30: 400 mg q24h | | 200 mg q24h (Post-dialysis administration on dialysis days) |
|  | Target:  *Pseudomonas* *aeruginosa*/central nervous system infection | - 30: 400 mg q8h (For an hour) | | < 30: 400 mg q12h | | 400 mg q24h (Post-dialysis administration on dialysis days) |
| Levofloxacin (LVFX) | | 500 mg q24h (For an hour) | 20-50: 500 mg × 1 dose, then 250 mg q24h | <20: 500 mg × 1 dose, then 250 mg q48h | | 500 mg × 1 dose, then 250 mg q48h |
| Azithromycin  (AZM) | | 500 mg  IV q24h (For 2 hours) | | | | |
| Teicoplanin  (TEIC) | | Day 1・Day 2 10 mg/kg q12h　**  (Over 30 minutes) | | | | |
| Vancomycin  (VCM) | | 25-30 mg/kg × 1 dose　*** (Over an hour) | | | | |

Subjects must be at least 15 years old and weigh at least 40 kg. Patients with muscular dystrophy and conditions that cause muscle loss, such as amputation of lower limbs, as well as obese patients (body mass index ≥30) were excluded due to difficulty in predicting pharmacokinetics. The Ccr estimate is most commonly used to adjust the approved dosage for renal impairment and evaluate renal function. Ccr estimate (mL/min) = (140 - age) × weight (kg) / 72 × (sCre + 0.2) (mg/dL), multiply by 0.85 for women.

*If the first dose was administered before dialysis, administration was avoided within 2 hours before dialysis.

**The maintenance dose was adjusted based on blood albumin concentration and trough values ​​in Therapeutic Drug Monitoring.

***The maintenance dose was adjusted using software based on a population analysis of Japanese subjects using PAT.

Ccr = Creatinine clearance; sCre = Serum creatinine; HD = Hemodialysis; IV = Intravenous; PAT = Practical Area under the time-concentration curve-guided Therapeutic drug monitoring.

Supplementary Table 2. Dosage of oral antibacterial agents administered to patients with renal impairment

| Drug | | Bioavailability | Ccr > 50 mL/min | Ccr 25-50 mL/min | Ccr 10-25 mL/min | Ccr 10 mL/min | HD |
| --- | --- | --- | --- | --- | --- | --- | --- |
| Amoxicillin  (AMPC) | | 90% | >30: 500 mg q8h | | 10-30: 250-500 mg q12h | 250-500 mg q24h | 250-500 mg q24h (Post-dialysis administration  on dialysis days) |
| Amoxicillin/clavulanic acid  (AMPC/CVA) | | 90%/60% | >30: 500*/125 mg q8h | | 10-30: 250-500*/125 mg q12h | 250-500*/125 mg q24h | 250-500*/125 mg q24h (Post-dialysis administration  on dialysis days) |
| Cefaclor  (CCL) | | 82.5% | 500 mg q8h | | | 500 mg q12h | 500 mg q24h (One of the dialysis days post-dialysis administration) |
| Ciprofloxacin  (CPFX) | | 60-80% | >30: 400 mg q12h | | <30: 400 mg q24h | | 200 mg q24h (Post-dialysis administration on dialysis days) |
| Levofloxacin  (LVFX) | | 99% | 500 mg q24h | 20-50: 500 mg × 1 dose, then 250 mg q24h | <20: 500 mg × 1 dose, then 250 mg q48h | | 500 mg × 1 dose, then 250mg q48h |
| Azithromycin  (AZM) | | 37% | 500 mg q24h | | | | |
| Clindamycin  (CLDM) | | 90% | 300 mg q8h | | | | |
| Metronidazole (MNZ) | | 100% | 500 mg q8h | | | 500 mg q12h | 500 mg q12h (One of the dialysis days post-dialysis administration) |
| Trimethoprim-sulfamethoxazole (TMP/SMX) | Target: general infections | 86-100% | >30: 2 tablets/dose q12h | | 15-30: 1 tablet/dose q12h | <15:  should not be administered | |
|  | Target: *Pneumocystis* pneumonia |  | >30: 5 mg/kg (TMP) q8h (e.g., for a body weight of approx. 50 kg, 3 tablets/dose q8h) | | 10-30: 5 mg/kg q12h (e.g., for 50 kg body weight,  3 tablets/dose q12h) | 5 mg/kg q24h** (e.g., for 50 kg body weight,  3 tablets/dose q24h | 5 mg/kg q24h** (Post-dialysis administration on dialysis days) (e.g., for 50 kg body weight,  3 tablets/dose q24h) |

Subjects must be at least 15 years old and weigh at least 40 kg. Patients with muscular dystrophy and conditions that cause muscle loss, such as amputation of lower limbs, as well as obese patients (body mass index ≥30) were excluded due to difficulty in predicting pharmacokinetics. The Ccr estimate is most commonly used to adjust the approved dosage for renal impairment and evaluate renal function. Ccr estimate (mL/min) = (140 - age) × weight (kg) / 72 × (sCre + 0.2) (mg/dL), multiply by 0.85 for women.

*AMPC/CVA (500/125 mg) not approved, AMPC/CVA (250/125 mg) + AMPC 250 mg. **Not recommended, but when used. Ccr = creatinine clearance; sCre = serum creatinine; HD = hemodialysis.

Supplementary Table 3. Reasons indicated for not switching to oral treatment

| Total patients | Control group (n = 23) | PBPM group (n = 23) |
| --- | --- | --- |
| No clinical improvement | 3 (13.0) | 5 (21.7) |
| No susceptibility to oral antimicrobials | 3 (13.0) | 2 (8.7) |
| Oral route uncompromisable ^a^ | 3 (13.0) | 11 (47.8) * |
| Intubation | 1 (4.4) | 3 (13.0) |
| Others | 14 (60.9) | 7( 30.4) |

As applicable, more than one reason could be checked per patient. The data are expressed as numbers with percentages in parentheses. Categorical variables were compared using Fisher’s exact test. **P* < 0.05, significantly different from the control group. ^a^Oral route uncompromisable was defined as fasting for more than 3 days, gastrointestinal bleeding, or frequent watery diarrhea. PBPM = protocol-based pharmacotherapy management
