# Supplementary material for: Efficacy of protocol-based pharmacotherapy management in switching of antibiotic administration routes and dose adjustment based on renal function: a before-after study
Source: J Pharm Health Care Sci. 2025 Dec 24;11:113. doi: 10.1186/s40780-025-00512-8 (PMC12729801; doi:10.1186/s40780-025-00512-8)
Supplement: Supplementary file 2 — Supplementary Material 2 [file 40780_2025_512_MOESM2_ESM.pptx]

## Slide 1
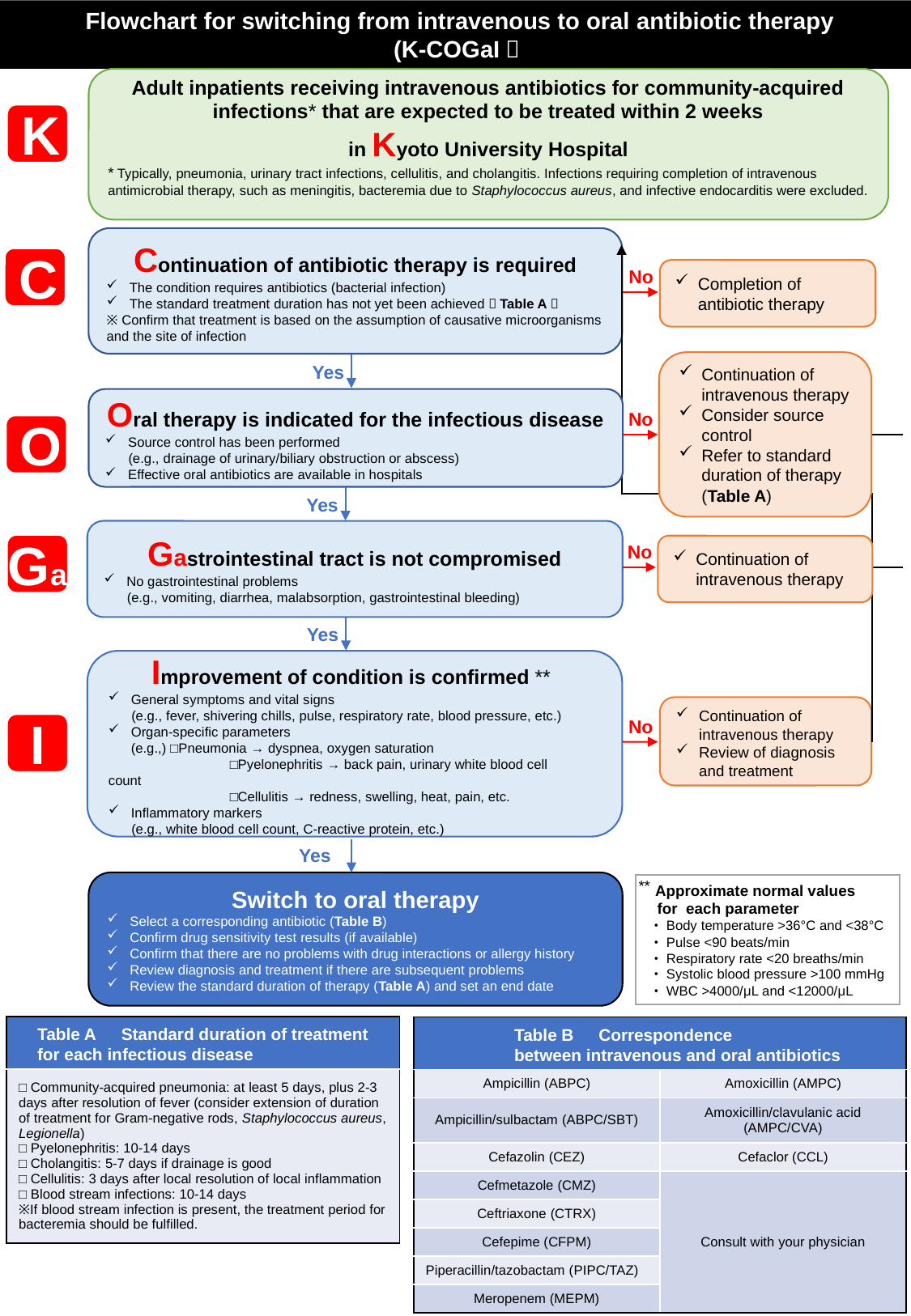

Flowchart for switching from intravenous to oral antibiotic therapy
(K-COGaI）
Adult inpatients receiving intravenous antibiotics for community-acquired infections* that are expected to be treated within 2 weeks
in Kyoto University Hospital
* Typically, pneumonia, urinary tract infections, cellulitis, and cholangitis. Infections requiring completion of intravenous antimicrobial therapy, such as meningitis, bacteremia due to Staphylococcus aureus, and infective endocarditis were excluded.
K
Continuation of antibiotic therapy is required
The condition requires antibiotics (bacterial infection)
The standard treatment duration has not yet been achieved（Table A）
※ Confirm that treatment is based on the assumption of causative microorganisms and the site of infection
C
No
Completion of antibiotic therapy
Continuation of intravenous therapy
Consider source control
Refer to standard duration of therapy (Table A)
Yes
Oral therapy is indicated for the infectious disease
Source control has been performed
 (e.g., drainage of urinary/biliary obstruction or abscess)
Effective oral antibiotics are available in hospitals
No
O
Yes
Gastrointestinal tract is not compromised
No gastrointestinal problems
 (e.g., vomiting, diarrhea, malabsorption, gastrointestinal bleeding)
Ga
No
Continuation of intravenous therapy
Yes
Improvement of condition is confirmed **
General symptoms and vital signs
 (e.g., fever, shivering chills, pulse, respiratory rate, blood pressure, etc.)
Organ-specific parameters
(e.g.,) □Pneumonia → dyspnea, oxygen saturation
	□Pyelonephritis → back pain, urinary white blood cell count
	□Cellulitis → redness, swelling, heat, pain, etc.
Inflammatory markers
 (e.g., white blood cell count, C-reactive protein, etc.)
Continuation of intravenous therapy
Review of diagnosis and treatment
No
I
Yes
**
Switch to oral therapy
Select a corresponding antibiotic (Table B)
Confirm drug sensitivity test results (if available)
Confirm that there are no problems with drug interactions or allergy history
Review diagnosis and treatment if there are subsequent problems
Review the standard duration of therapy (Table A) and set an end date
 Approximate normal values
 for each parameter
　・Body temperature >36°C and <38°C
　・Pulse <90 beats/min
　・Respiratory rate <20 breaths/min
　・Systolic blood pressure >100 mmHg
　・WBC >4000/μL and <12000/μL
| Table A　Standard duration of treatment for each infectious disease |
| --- |
| □ Community-acquired pneumonia: at least 5 days, plus 2-3 days after resolution of fever (consider extension of duration of treatment for Gram-negative rods, Staphylococcus aureus, Legionella) □ Pyelonephritis: 10-14 days □ Cholangitis: 5-7 days if drainage is good □ Cellulitis: 3 days after local resolution of local inflammation □ Blood stream infections: 10-14 days ※If blood stream infection is present, the treatment period for bacteremia should be fulfilled. |
| Table B　Correspondence between intravenous and oral antibiotics | |
| --- | --- |
| Ampicillin (ABPC) | Amoxicillin (AMPC) |
| Ampicillin/sulbactam (ABPC/SBT) | Amoxicillin/clavulanic acid (AMPC/CVA) |
| Cefazolin (CEZ) | Cefaclor (CCL) |
| Cefmetazole (CMZ) | Consult with your physician |
| Ceftriaxone (CTRX) | |
| Cefepime (CFPM) | |
| Piperacillin/tazobactam (PIPC/TAZ) | |
| Meropenem (MEPM) | |
